# Advancing Innovation in Medical Presentations: A Guide for Medical Educators to Use Images Generated With Artificial Intelligence

**DOI:** 10.7759/cureus.74978

**Published:** 2024-12-02

**Authors:** Mhd Firas Safadi, Obada Zayegh, Zakaria Hawoot

**Affiliations:** 1 Department of General and Visceral Surgery, Annaberg Teaching Hospital, University of Leipzig, Annaberg-Buchholz, DEU; 2 College of Medicine and Health, University of Exeter, Exeter, GBR; 3 Department of Neurology, Gummersbach Teaching Hospital, University of Cologne, Gummersbach, DEU

**Keywords:** ai-generated images, artificial intelligence, medical education, medical lectures, medical presentations, medical storytelling, multimedia learning theory, multimedia principles, multimedia theory, powerpoint presentation

## Abstract

Multimedia learning theory suggests that the use of images in the classroom enhances both cognitive and constructive learning. Finding the appropriate images for medical presentations can be challenging due to copyright and privacy issues. Artificial intelligence (AI) image generation is a newly available technology that offers a great advantage to medical educators. This technical report presents a robust guide for beginners to learn how to effectively use AI to generate images relevant to their presentations using free tools. The 12 guide tips include adhering to multimedia theory, using cost-effective resources, recognizing the possibilities, learning the technique, mastering the jargon, expanding the natural language, using prompt templates, creating stories, employing humor, avoiding perfection, exploiting the versatility of applications, and recognizing the limitations and ethical issues. By working through the article and the supplementary material, readers will be able to produce images similar to those provided in the article and use them effectively in their medical presentations.

## Introduction

The multimedia education theory suggests a synergistic effect between words and images, with the latter found to stimulate pictorial memory and enhance both cognitive and constructive learning [1]. The inclusion of media transforms medical presentation into a more efficient and enjoyable tool [2]. Images can also brighten dull topics and promote the relevance of the lecture content [3].

Careful selection of media for educational presentations poses significant challenges in different aspects [2]. Creative materials are commonly copyrighted, can be reuse-restricted, or require time-consuming permissions [3]. Stock images may be unaffordable for medical educators in developing countries, and they may have limited representation of certain ethnicities, age groups, or situations [4]. Finally, using photos of real patients may elicit ethical and legal issues, since healthcare workers may lack understanding of the consent process and ethical implications when reusing digital images [5].

The neoteric advancements in artificial intelligence (AI) introduced text-to-image (TTI) models capable of creating high-quality images for various uses, including medical education [6]. The availability of such tools at the educator’s fingertips overcomes many of the challenges for several reasons: (1) the desired images are accessible without the need for tedious search; (2) the process is cost-effective; (3) the end product shows high flexibility to refine the style, lighting, and characters’ ethnicity, which renders the educational content more culture-sensitive and setting-appropriate; and (4) the produced images override many copyright issues with fewer restrictions [4,7]. Indeed, the use of AI-generated images “… could alleviate concerns surrounding copyright infringement or patient privacy that are inherent in using clinical photos” [8].

In the last months, we had the opportunity to work with many of the available tools and employ the produced images for various purposes in medical education. In this article, we introduce 12 practical tips for those who want to use AI-generated images in their medical presentations. The tips are aimed at novice-level users who need a basic guide to break the ice. Following the article, we provide additional material that enables readers to produce the same high-quality images used in this article to elevate their medical presentations at no cost.

## Technical report

Keep multimedia principles in mind

Inappropriate use of images in medical presentations can distract the learners and impede the learning process. This use is governed by definite principles that should be regarded during the planning phase and applied in any presentation [9,10]. Therefore, a solid comprehension of multimedia educational theories and cognitive science helps to sidestep pitfalls that hinder multimedia-based learning. Mayer explained 12 principles that should underlie the use of multimedia in education, three of which are particularly relevant to AI-generated images in medical presentations [9].

According to the coherence principle, your generated image should be relevant and support the educational material. Random or decorative images may distract the learners and impact the learning process. The second rule is implied by the spatial contiguity principle, which states that the image should be placed next to the corresponding text to build a visual connection. This is especially important when you use more than one image on the same slide, where you have to allocate the appropriate text to the relevant image. According to the third principle, it is even better to minimize or eliminate the use of text in your slides in a narrated presentation, as the redundancy principle states [9]. This is a common mispractice among medical educators, as cognitive gain will be compromised when the learners read and listen at the same time. Therefore, it is important to avoid a large bulk of text on the slides and to present the information using short bulleted sentences.

Medical educators should be aware of all the principles of multimedia use before they add images to their presentations, whether original illustrations or those created using AI. Keep your focus on producing an experience that is learner-centered rather than technology-centered [10], and plan no more than one or two images on every slide with a minimal amount of text [1].

Think cost-effective

There are currently many available TTI models, such as DALL-E, Stable Diffusion, Midjourney, Adobe Firefly, Google ImageFX, Canva, Lexica, Parti, and Imagen [11,12]. One may be tempted to adopt a subscription-based model that can yield amazing visual outcomes. Although most of the services that provide high-quality, high-volume images are subscription-based, many of them also offer free plans. To generate the images used in this article, we used the free plan of Image Creator from Microsoft (Redmond, WA) [13]. The plan offers a daily number of images to use even for commercial purposes. With such tools, you can generate amazing images for your presentation at no cost, which shows one major benefit of TTI models [14].

Of course, subscription-based applications may include other advantages such as creating a larger number of images, having more control over details, obtaining higher resolution, generating images based on uploaded photographs, and making changes to specific elements in a given image [15]. However, it is always wise to start practicing with a free service before considering an upgrade. The few examples included in this article showcase the huge capabilities of free AI models if one learns how to employ them appropriately and productively.

Embrace the plethora of options

**Figure 1:**
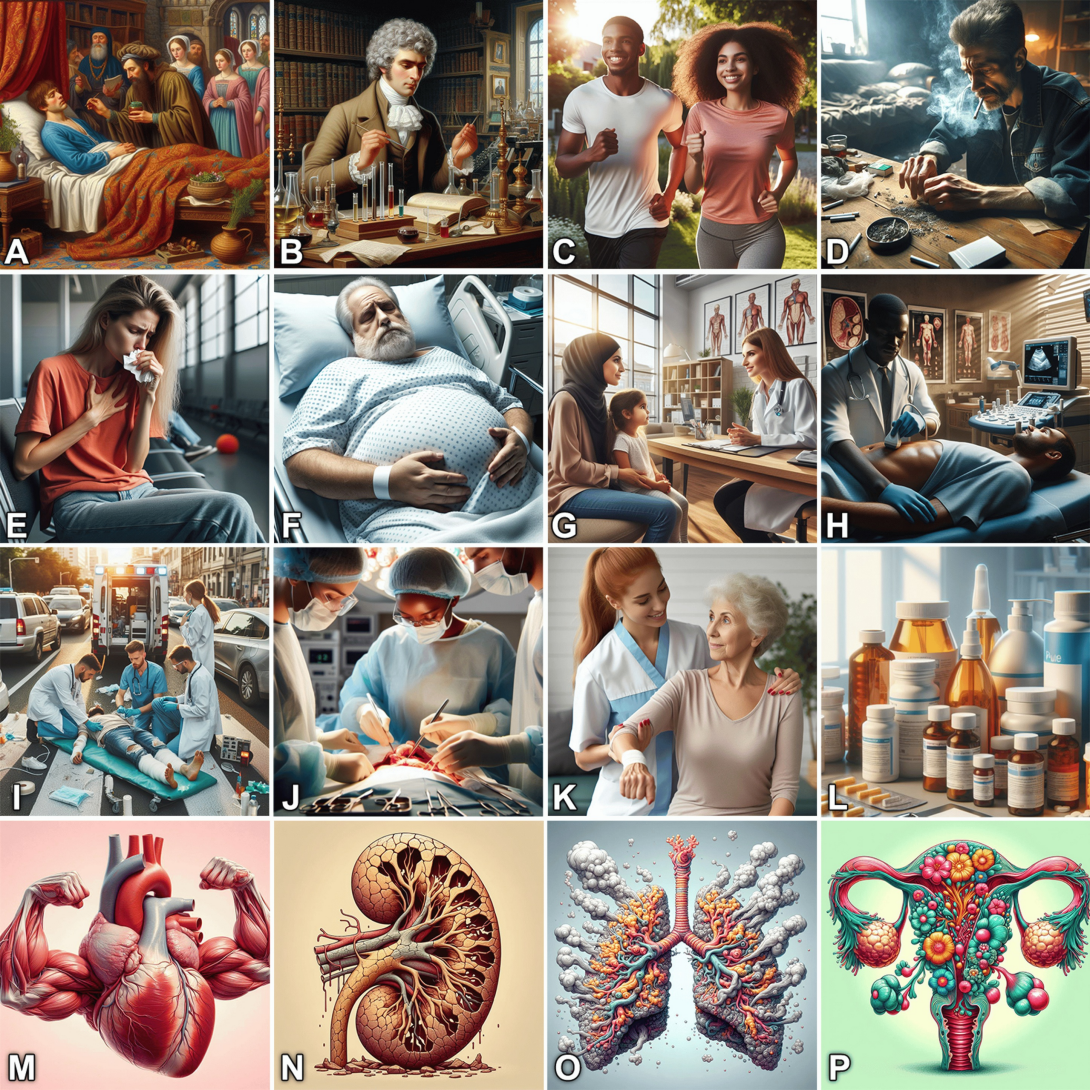
Examples of the wide spectrum of images that can be easily generated using the available artificial intelligence (AI) tools. The image may include historical scenes (A) or scientists (B); personal healthy habits (C) or unhealthy behavior (D); medical conditions such as pneumonia (E) or intestinal obstruction (F); medical practice such as history taking (G) or bedside tests (H); therapeutic interventions such as trauma life support (I), surgical operations (J) or physiotherapy (K); various medication packages (L); and symbolic anatomic illustrations of various organs, including the heart (M), kidney (N), lungs (O), and uterus (P). Images were generated using a free source: Image Creator from Microsoft [13].

Generative AI can offer medical educators a huge capability and flexibility to produce a wide spectrum of images and illustrations. Figure 1 shows just a few examples of AI-generated images that can be used in everyday medical presentations. Note the flexible control over the characters’ age, ethnicity, appearance, clothing, expressions, attitude, and image context, which overcomes many drawbacks of stock images [4].

Here are the prompts that were used to generate the images in Figure 1: (A) oil painting of a portrait from the Middle Ages showing a patient is lying on a bed in his room and a doctor treating him with herbal medicine, with few worried family members in the background, rich, colorful details, wide angels, furniture, and background of the Middle Ages, medium lighting. (B) Painting of a doctor from the eighteenth century doing an experiment in his laboratory, testing tubes, various scientific instruments, old books, background library, rich details, low contrast, soft light, low angle shot. (C) Young African man and Hispanic woman running in a garden under sunlight with sport t-shirts, happy atmosphere, photorealism, high resolution, precise details. (D) A sick Caucasian man in his forties sitting at a wooden table and smoking with burned cigarettes in the tray, messy room, low angle shot, photorealism, high resolution, precise details, cinematic lighting. (E) Medium shot of a distressed white woman aged forty with long blonde hair seated in the well-lit atmosphere, wearing an orange t-shirt and jeans and sitting in a hospital waiting hall, she cradles her chest with her left hand, attempting to stifle a relentless cough, she clutches a paper tissue in the other hand, stunning photorealism, eye level, natural internal light. (F) A 65-year-old Caucasian overweight man lies in a hospital bed, wears a hospital dress under white sheet, his both arms are on his big belly, he is distressed and depressed, photorealistic, diagonal view, indoor lighting, medical equipment background. (G) A Middle Eastern woman with her little daughter having a conversation with a European female doctor in her office, photorealism, wide angle, bright lighting, rich office background, medical illustrations on the walls. (H) An African doctor performing ultrasound examination of the belly of a young Asian man in a hospital setting, photorealism, high camera angle, hospital equipment in the background, radiological illustrations on the walls, cinematic lighting, high resolution. (I) A medical team treating a patient with a road traffic accident on a street in a busy city with an ambulance in the background, critical situation, photorealism, high camera angle, daylight, rich details. (J) A team of three surgeons performing a surgery in the operation room, realistic photo, bright light, wide angle, rich details. (K) A white female physiotherapist with red hair helping a senior woman in training, realistic photo, high camera angle, precise details. (L) A realistic photo of different medications including packages, tablets, and creams on a table in a doctor's office, indoor lighting, high resolution. (M) A surrealistic drawing of a strong human heart, exaggerated anatomical illustration, light pink background. (N) A surrealistic illustration of a human kidney, destroyed with multiple cracks and fissures, beige background. (O) A surrealistic drawing of lungs made of fume and aches, exaggerated anatomical illustration, light grey background. (P) A surrealistic drawing of the uterus and ovaries flowering in shining colors, exaggerated anatomical illustration, light green background.

The illustrated examples are far from being representative of the comprehensive possible contexts, as educators can create thousands of other ideas for illustration purposes. Some other examples may include metaphorical representations of the concepts, abstract medical illustrations, or design blocks as part of combined illustrations that include mnemonics, mind maps, or infographics [3,14].

Different TTI models can be used for focused generative goals when one chooses to use a subscription-based model. For example, Midjourney is more socially integrated, while Stable Diffusion is more customizable [16]. Medical educators may use their imagination to hit upon unusual ideas that produce extraordinary images for their illustrations, which would eventually enhance the learners' experience and engagement.

Learn the art of image generation

Machines follow the given prompt to generate the sought images. A "prompt" is defined as the input instructions you write in the command box to obtain the desired image. It may range from one word to one sentence up to a very detailed visual description of the desired image [7].

Analysis of successful prompts revealed that generating images using TTI models bears close resemblance to fundamental principles used in photography education. A creative process was proposed by Dehouche et al. comprising three elements: (1) mise-en-scene: the objects, environment, and characters in the image frame (what); (2) dispositif: the technique and processing of the image and how the items should look (how); and (3) cultural object: includes other determinants of image and its purpose like specific time of history or purpose (why) [12].

Another art framework was mentioned in *A Traveler’s Guide to the Latent Space* and can be applied to generate art for medical education [17]. The framework consists of five consequent elements in a fixed order (medium, subject, artist, details, and image repository support), which will yield a quality image if applied competently. Following its development, the latter framework was reckoned by the DALL-E prompt book as an effective approach [17,18]. As a novice user, you can start with the simplest approach that suits your needs. There has also been a consensus among creators that the prompt is made up of a skeleton with specific essence parts. The proficient selection of words in these parts can amplify the quality of the resulting images. As commonly referred to, "prompt modifiers" were presented in great detail in the work of Oppenlaender [19].

Prompt engineering is a rapidly growing topic but is not challenging to learn and often follows the rule of trial and error [19]. As a novice user, you can start practicing immediately using tips provided in our guide. One important tip in the art of image generation is to start with a short prompt, including only essential elements. Subsequently, craft your prompt in iterative stages to obtain a satisfactory outcome, while avoiding complexity that may lead to unexpected results [20]. For example, to generate Figure 1K, we started with the short prompt “physiotherapist helping a senior woman” and then enhanced the image to add more details.

Learn the artistic jargon

Learning the specific terms that the TTI models understand is a key requirement for producing the desired images, as these models would respond to your prompt according to the words that you use. Similar to photography, where fine-tuning camera settings can transform your photos from simple capture to a piece of art, using advanced artistic jargon in your prompt will yield the outcomes you desire [21]. In prompt writing, using the appropriate jargon would yield the desired artistic style of your images.

Table 1 shows many elements that you can include to control the style, paint, camera, lighting, people, and location within your masterpieces. Many of these keywords were already used to produce the images included in this article. To improve the productivity of your prompts, we recommend that you carefully inspect the prompts attached to the images and learn how to effectively apply the jargon in image production. Note that the included material is only a place to start, and you may view plenty of online resources for more visual feeding [22]. Additionally, you are encouraged to use your imagination and extend the content of this table as necessary, adapting it to your specific educational context and needs.

**Table 1:** Helpful prompt elements for generating images for medical presentations. To become familiar with the possible results, the reader is encouraged to test various styles.

Element	Examples
Style	photorealism, surrealism, impressionism, expressionism, minimalism, digital art, cartoon, Disney style, Pixar style
Paint	hand drawing, pencil sketch, charcoal, pastel, watercolor, oil paint, Chinese brush, digital paint
Camera	eye level, low angle, high angle, front view, back view, side view, diagonal view, close up, macro shot, wide shot
Lighting	indoor, outdoor, bright, dark, daylight, soft light, hard light, backlight, cinematic light, low-key light
People	baby, child, adolescent, young, adult, senior, African, Arab, Asian, Caucasian, Hispanic, (specific age), (specific country)
Location	living room, garden, street, doctor office, emergency department, waiting hall, hospital corridor, hospital ward, operating room, intensive care unit

Elements in your image can be produced in different styles by changing no more than one word in your prompt. A thermometer, for example, can be real or a drawing. When you imagine the outcome you desire, try to learn how to express the style you want in words by knowing more about the artistic jargon [21]. In the same way, learning photography terminology can transform your images from simple capture to a magnified macro image expressing a deeper focus on the image details [12]. As a practical application, the artistic appearance of your images will change if you mention specific eras when lecturing about the history of medicine.

Expand your natural language

Mastering the natural language in which you write your prompts engenders a unique output that reflects your educational needs [11]. Research revealed that images generated with more linguistically precise prompts were higher in quality. Using more unique vocabulary will trigger the models to produce lone results. In addition, the context and sequence of your prompt’s words will influence the process. Therefore, the grammatical and linguistic features you choose for your prompt will be reflected in the final image [23].

Creators frequently employ thesauri to augment their lexical exploration, uncovering distinctive synonyms that TTI can intricately weave into their artistry. Some artists share their linguistics expertise on domain-specific blogs to gain collective opinions and enrich their natural language reserves. To the best of our knowledge, we do not recognize an established linguistic resource for employing art in medical education. However, expanding natural language skills was emphasized in the prompt analysis as a key to exceeding the average outcome, which can be essential if you are using a second language to write your prompts. [11]. This should induce future initiatives for collaborative platforms dedicated to AI-generated art in medical education.

An additional tip is to ask any AI model to write the prompt for you: “I need a detailed 50-word prompt to produce an image of a young boy being examined by a female doctor.” Just copy the prompt that you get in the field of image generation and add the final touches to get outstanding results. Also, some TTI models will provide an automated description of the generated image, which serves as a robust ground for further prompt refining.

Use prompt templates to achieve consistency

Countless outcome possibilities can disrupt the consistent style of the presentation. Diversifying the styles and including extravagant details will rather distract the learner and increase cognitive overload [24]. Nevertheless, some simple techniques can help you create consistent images, hereby granting your presentation a unique style. A "prompt template" is a piece of text that describes the outcome image with slots kept for varying elements. When well-crafted, templates present a powerful tool that can help you construct a consistent style for your presentation, offer the potential to create shareable style prompts, and reflect your artistic will [11].

**Figure 2:**
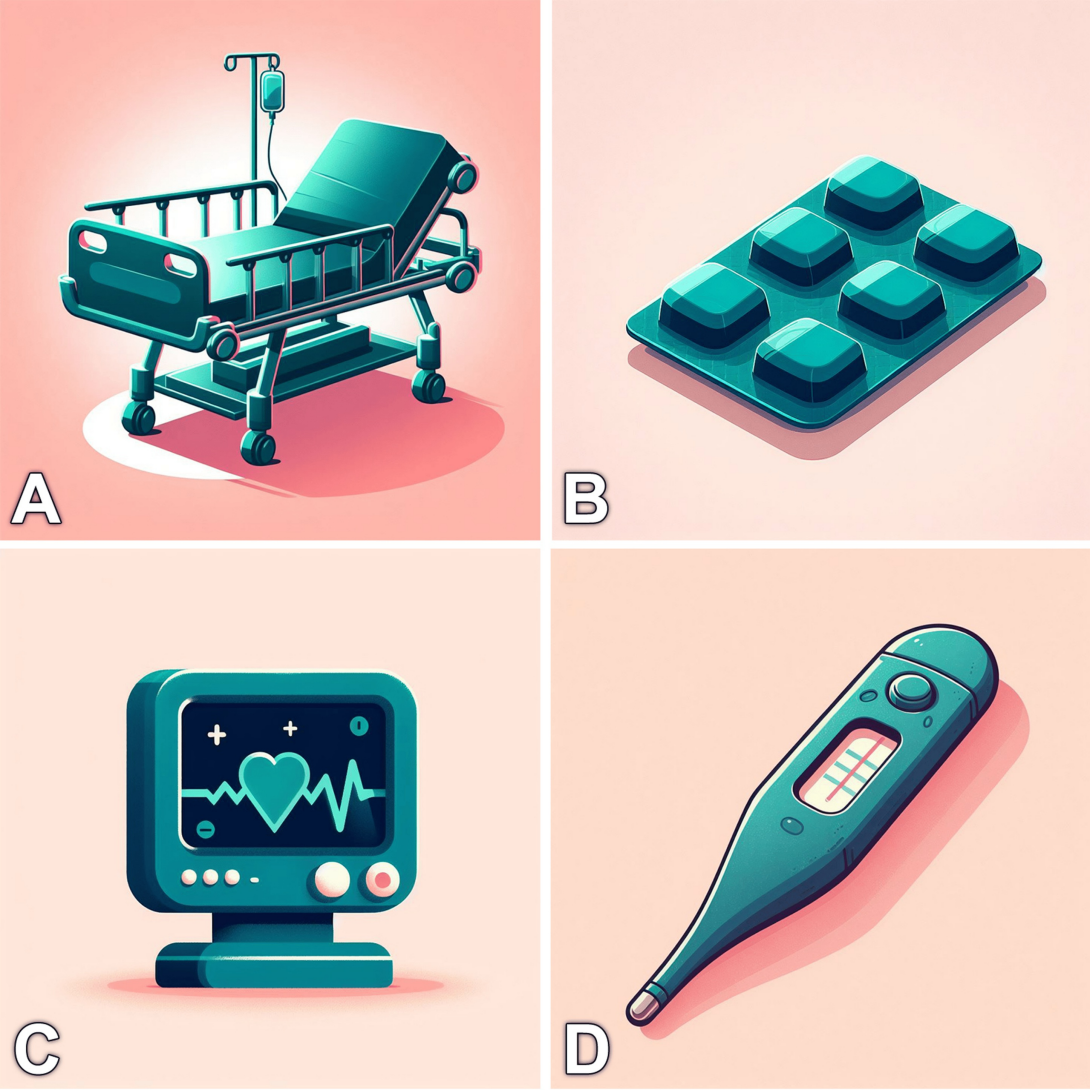
Using prompt templates to create consistent medical images. Creating illustrations of a hospital bed (A), pills (B), monitor (C), and thermometer (D) that have a consistent style and color palette using a prompt template: “A Pixar style illustration of a dark teal [DESIRED OBJECT] on a light pink background.” The description of the desired object can be added between the brackets. The reader can tailor the technique to generate thousands of styled icons or objects Images were generated using a free source: Image Creator from Microsoft [13].

One possible application of this technique is shown in Figure 2. Here, we used a unified template as a description skeleton and left placeholders for the desired objects. Consequently, we were able to produce various images that have the same artistic style and can be used throughout the presentation without breaching the slides’ harmony. Another alternative method is to ask the model to present the desired objects in a 2 x 2 or 3 x 3 grid. However, the individual elements may be less customizable compared to the template technique. Maintaining consistency bestows a professional look at your presentation, minimizes disruption, and keeps the learners involved [24]. Furthermore, the technique enables you to present your images using desired colors in consistency with the global design of your presentation. According to the color theory, adhering to a definite palette is considered a good practice when generating scientific figures [25].

Here are the prompts that were used to generate the images in Figure 2: (A) a Pixar-style illustration of a dark teal hospital bed on a light pink background. (B) A Pixar-style illustration of a dark teal medication package on a light pink background. (C) A Pixar-style illustration of a dark teal medical monitor on a light pink background. (D) A Pixar-style illustration of a dark teal table of medical instruments on a light pink background.

Image generation is an evolving field with new technologies born round-the-clock. Some models can now analyze the prompt and the resulting image history to illustrate the relationship between key changes in the prompt and how they are reflected in your images. This tool would consequently help in planning changes for accurate outcomes [26]. Another tool can retrieve image-prompt pairs and use them to suggest relevant keywords and their impact on your image to create an interactive experience [27]. Make sure you keep yourself updated about new technologies and tools if you want to stand out with your skills.

Be creative in storytelling

Case-based learning uses real-life scenarios to connect theory to practice, thereby enhancing basic understanding besides improving treatment outcomes [28]. Educators can now generate multiple photos of patients and staff in different situations to create interactive cases, which were fraught with ethical issues or expensive artist costs in the past [5,14]. Use the TTI models to generate impressive visual scenarios incorporated into digital storytelling.

**Figure 3:**
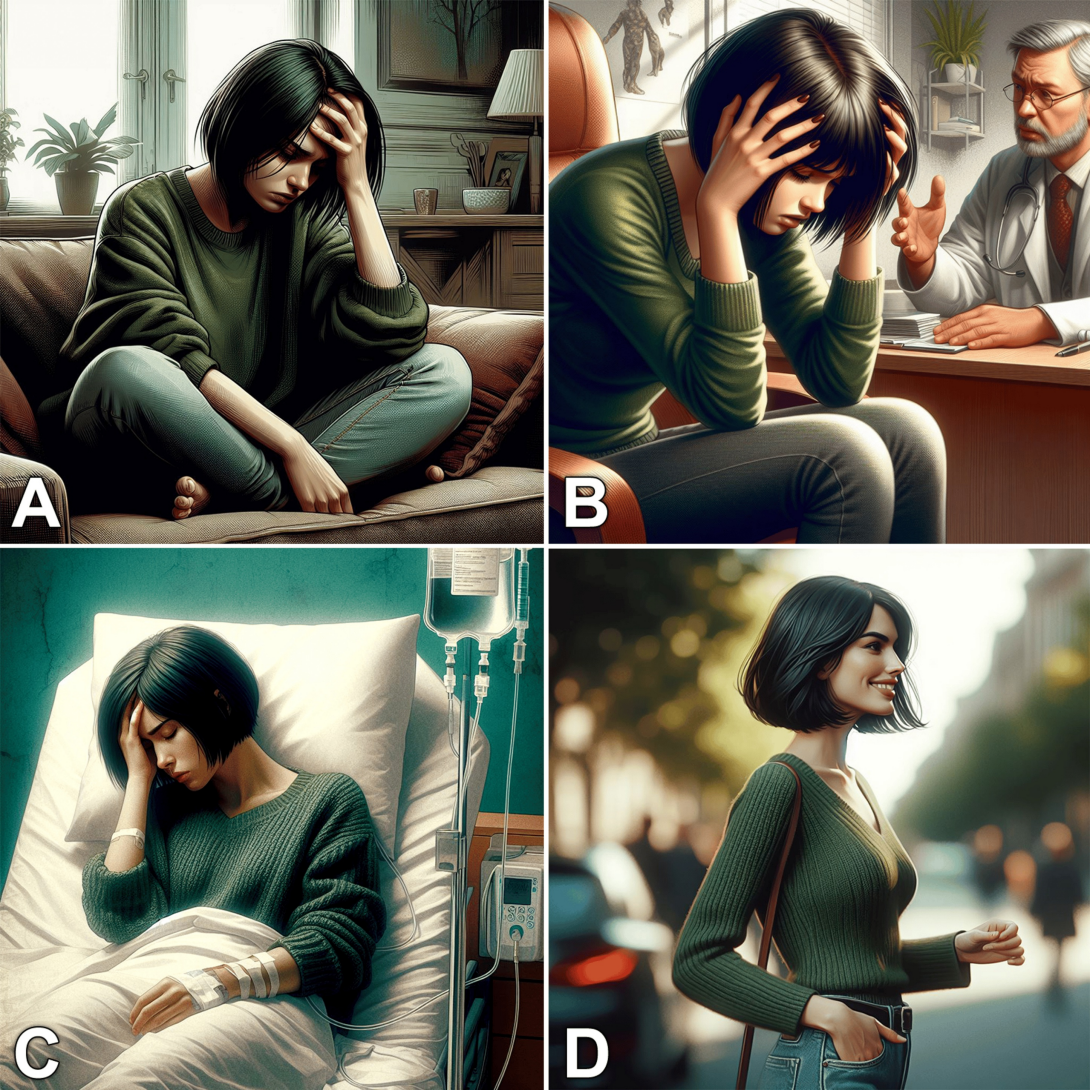
Using generative artificial intelligence to produce visual medical scenarios. These images were produced separately using the following prompt template: “A stressed 30-years-old Caucasian woman, exhausted with headache, has straight short black hair, wears a green sweater and jeans, [DESIRED DETAILS], digital art, rich background details.” In the brackets, we added the desired description of the image context: at home (A), at the doctor’s office (B), in the hospital (C), and after successful treatment (with a slight change of the final prompt) (D). The characters may not be 100% identical, but the images do tell a story and the nuances may not be noticeable unless the images are displayed on one slide. You can also ask for a grid of multiple images within one prompt, but you will have less control over the details of each image. Images were generated using a free source: Image Creator from Microsoft [13].

Figure 3 shows how you can create a scenario of a female patient with headaches. Simply create a template with a detailed description of your desired character, then change the setting in every prompt as needed. Include the images on your slides, insert further radiologic and clinical images in between, let your virtual hero accompany the audience, and engage them throughout the presentation. Provoking students’ emotional reactions and stimulating their interaction will highly enhance their learning experience [29].

Here are the prompts that were used to generate the images in Figure 3: (A) a stressed 30-year-old Caucasian woman, exhausted with headache, has straight short black hair, wears a green sweater and jeans, sits on a couch in the living room, digital art, rich background details. (B) A stressed 30-year-old Caucasian woman, exhausted with headache, has straight short black hair, wears a green sweater and jeans, sits on a chair in a doctor's office and explaining her problem to the doctor, digital art, rich background details. (C) A stressed 30-year-old Caucasian woman, exhausted with a headache, has straight short black hair, wears a green sweater and jeans, lies in a hospital bed under a white sheet with an infusion bag next to the bed, digital art, rich background details. (D) A 30-year-old Caucasian woman, has straight short black hair, wears a green sweater and jeans, walks in the street, glad, side diagonal view, digital art, blurred background details.

If you plan to produce many images using this technique, it is important to clearly outline your story before turning to the AI model. Furthermore, you may need to test frequently and make slight adjustments every time to achieve the desired image consistency. Recently, many subscription-based models enable the user to create multiple images based on a predefined character [15], which can hugely simplify the process of creating serial images for storytelling purposes.

Let your humor shine

Humor has been long known to enhance learning in the classroom [30]. The instructional humor processing theory states that humor stimulates the students to analyze the necessary elements for message elaboration, which in turn facilitates learning by boosting students’ attentiveness and making the information memorable [31]. Using the various techniques described in the above sections, the medical educator can employ TTI models to generate humorous images that can be incorporated into the presentation, further enhancing the learners’ experience.

**Figure 4:**
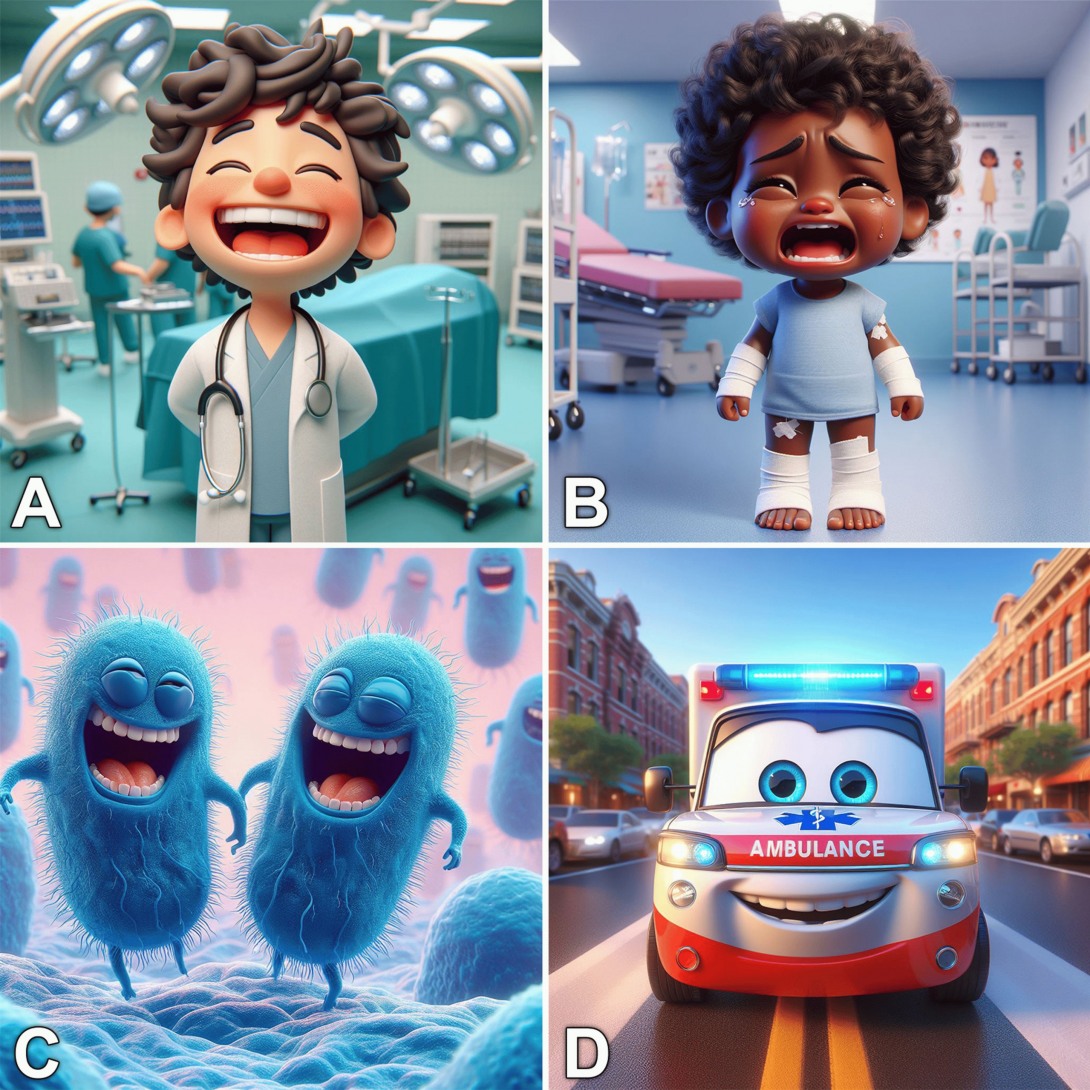
Using generative artificial intelligence to produce humorous medical images. This illustration shows several examples of humorous images that can be easily generated using AI: (A) medical staff, (B) pediatric patient, (C) resistant bacteria, and (D) ambulance. Images were generated using a free source: Image Creator from Microsoft [13].

As one variant of visual humor, funny cartoon images or three-dimensional designs can particularly add intense emotions to the lesson, thereby acting as a strong stimulus to enhance student interaction and banish boredom [3]. As Figure 4 shows, you can create comic images with almost no skills and proudly add them to your show to "make them laugh" [31]. Indeed, obtaining such images in the previous era was not possible without the help of a talented and dedicated artist, obviously with additional time and cost [4,7]. Obviously, you should always consider the relevant and appropriate context before incorporating such images in your presentations.

Here are the prompts that were used to generate the images in Figure 4: (A) 3D cartoon of a funny surgeon with curly hair standing in the operating room with a hysteric laugh and closed eyes, wide shot, diagonal view, high resolution, context background. (B) 3D cartoon, funny crying two-year-old dark-skinned girl with curly dark hair, standing in the emergency department, plaster patches on her arms and legs, wide shot, front view, high resolution, context background. (C) 3D cartoon, two funny blue bacteria, laughing with devil face expressions and sharp teeth, walking inside a microscopic tissue environment, side view, high resolution, context background. (D) 3D cartoon of a funny ambulance car in the style of Cars movie smiling with trust with a blue light above within a street in a busy city, wide shot, front view, high resolution, context background.

Evade the trap of perfection

Generating images may exhaust your time if you are too critical about non-key details. TTI models process a prompt that consists of a few words to generate a whole image. Many details may be filled in the output images without being described in the prompt. The interaction between the prompt’s words can produce an unpredictable conclusion. Unless it poses an ethical problem or has significant implications on learning, try to embrace the imperfection and even use it for your own benefit. This was even acknowledged by an artist who reported that he “…hunted for interesting imperfections instead of avoiding glitches” [11].

Remember also that the images are created for educational purposes and not for a gallery. Thus, including many details in the image can increase the cognitive burden, distract the learners, and impede effective learning [24]. Do not waste much time trying to get perfect results if the produced images already convey the message that you want to deliver.

Exploit the versatility of applications

Images generated using TTI models for medical presentations may have a far wider array of applications other than mere illustration. Images can challenge topics of attitudes and professionalism in the medical practice. Through art-enriched learning, you can engage your students in the creation or reflection on artworks [32]. Use the images that illustrate life experiences to empower the students’ capabilities of self-awareness, emotional processing, and debriefing. Such activities help develop better emotional intelligence, which was shown to achieve greater success in nursing school, enhance adaptation to challenges, and reduce the chances of burnout [32].

The generated images may be also employed for testing in written or oral examinations. Building professional identity and challenging the public image of health careers can also be supported by the proper use of images. The successful application was found to encourage career decisions and lessen drop-outs and recruitment difficulties [32]. All of these applications can be reinforced by the variety, flexibility, and ease of use offered by generative AI.

Recognize ethicalities and limitations

It is imperative that you adhere to moral, ethical, and legal principles when using AI-generated images. Familiarize yourself with the "Terms and Conditions" or "Code of Conduct" of any application before you sign up to use. For instance, creating disturbing or offensive content that encourages violence or abuse may result in your account being suspended. Additionally, you still need to acknowledge the use of AI for transparency purposes as an ethical best practice [5]. When using TTI-generated images for your medical presentations, we recommend adding a short notice in the caption referring to the used platform. Note how we acknowledged the images in this article.

Although many works highlighted the ability of AI models to generate copyright-free images that do not need patient’s consent [14], the available tools are not yet capable of generating complex medical images, as they may misinterpret or not understand medical jargon. Images simulating medical conditions necessitate huge refining and sometimes editing to satisfy the experts’ description of clinical signs, deformities, injuries, or neoplasms [6,33,34]. Furthermore, the available models are unable to generate precise anatomical or surgical illustrations. Even the production of a normal chest X-ray or electrocardiogram failed due to model limitations [35]. Finally, most applications will block distressing images or striking features when you include words such as "wound" or "blood" in your prompt. You may turn around some limitations and provide an alternate description to avoid the flagged words. For example, "An old man coughing and holding a red-stained handkerchief" may yield an illustration of hemoptysis.

Copyright issues present another important limitation that should be considered. The AI models are trained on thousands of real, sometimes copyrighted, photographs and the generated images are more or less based on them. As a result, there has been some controversy over the ownership and the reuse of the generated images [6]. Additionally, due to the availability of data that AI machines were trained on, there has been some reported latent bias in representing genders and races in the output images. Images were sometimes described as emphasizing false stereotypes about health professions [32]. The conscious use of TTI models with appropriately orientated prompts can make the experience more inclusive and avoid unexpected marginalization [33].

## Discussion

The integration of AI-generated images into medical presentations offers a valuable tool for medical educators. The medical teacher can now create engaging and appealing presentations without the need for artistic expertise or significant costs. This technical report presents a frame of 12 tips to regulate and guide the use of AI-generated images in medical presentations, focusing on their advantages and applications and underscoring usage pitfalls.

The generated images empower educators to tailor visuals to precise educational contexts, which cannot be matched by stock images. The ability to control further details such as age, ethnicity, and surroundings offers an unlimited potential for customization, which helps bridge the gap between abstract concepts and real-world applications [4]. Free TTI models, such as those utilized in this article, allow even novice educators to experiment and create professional-grade visuals.

An essential and challenging aspect of leveraging AI-generated images lies in the art of prompt engineering and stylistic consistency. Crafting effective prompts requires not just linguistic precision but also an understanding of artistic and contextual elements that the models understand [12,17]. The mentioned techniques enable medical educators to generate a series of cohesive images to employ them in storytelling and case-based learning while matching the design and color outputs to their needs and minimizing cognitive overload for learners [24].

On the other hand, educators must recognize the multimedia principles and adhere to them while using these tools to streamline the learning experience and avoid unnecessary distractions [9]. Additionally, the affordability of these tools raises the challenge of quality over quantity: without deliberate planning, the easy generation of images might lead to their overuse or misuse in presentations. Balancing the technical capabilities with thoughtful educational goals ensures that visuals remain tools for engagement rather than sources of distraction.

Despite its transformative potential, the use of AI-generated images in medical education is not without limitations. The inability of current models to reliably generate complex medical visuals underscores a possible technological gap [33,35]. Ethical use further demands transparency, especially concerning acknowledging the source of the images and respecting copyright [5,6]. These challenges highlight the need for educators to adopt a reflective and adaptive approach, ensuring that the integration of AI tools enhances learning while upholding professional and ethical standards.

## Conclusions

Creating stunning images is no longer limited to graphic designers. Any medical teacher can learn and master the art of AI image generation and put the results into practice. The applications of AI-generated images in medical presentations are not limited to the examples presented in this article, and there are endless possibilities for using artwork in education. We believe it is a skill that every medical educator should learn and master. The 12 tips we have presented in this article, together with the simple prompts provided with the images, are an excellent place for beginners to start.

Soon, the process of image generation will become even easier and faster. AI-generated images will surely find their way into academic medical publications, including journal articles and books. Because the technology is still relatively new, we wanted to shed some light on the possible ways to link AI images with medical education, and we believe that the ideas mentioned can inspire researchers in medical education to conduct in-depth future studies and explore further practical applications.
